# Selectively bred oysters can alter their biomineralization pathways, promoting resilience to environmental acidification

**DOI:** 10.1111/gcb.14818

**Published:** 2019-09-25

**Authors:** Susan C. Fitzer, Rona A. R. McGill, Sergio Torres Gabarda, Brian Hughes, Michael Dove, Wayne O'Connor, Maria Byrne

**Affiliations:** ^1^ Institute of Aquaculture University of Stirling Stirling UK; ^2^ Scottish Universities Environmental Research Centre Scottish Enterprise Technology Park East Kilbride UK; ^3^ School of Medical Sciences University of Sydney Sydney NSW Australia; ^4^ Hunter Local Land Services Taree NSW Australia; ^5^ New South Wales Department of Primary Industries Fisheries NSW Port Stephens Fisheries Institute Taylors Beach NSW Australia; ^6^ School of Life and Environmental Sciences University of Sydney Sydney NSW Australia

**Keywords:** aquaculture, calcification, carbon pathway, climate change, estuary, low pH, *Saccostrea glomerata*, selectively bred families, Sydney rock oyster

## Abstract

Commercial shellfish aquaculture is vulnerable to the impacts of ocean acidification driven by increasing carbon dioxide (CO_2_) absorption by the ocean as well as to coastal acidification driven by land run off and rising sea level. These drivers of environmental acidification have deleterious effects on biomineralization. We investigated shell biomineralization of selectively bred and wild‐type families of the Sydney rock oyster *Saccostrea glomerata* in a study of oysters being farmed in estuaries at aquaculture leases differing in environmental acidification. The contrasting estuarine pH regimes enabled us to determine the mechanisms of shell growth and the vulnerability of this species to contemporary environmental acidification. Determination of the source of carbon, the mechanism of carbon uptake and use of carbon in biomineral formation are key to understanding the vulnerability of shellfish aquaculture to contemporary and future environmental acidification. We, therefore, characterized the crystallography and carbon uptake in the shells of *S. glomerata,* resident in habitats subjected to coastal acidification, using high‐resolution electron backscatter diffraction and carbon isotope analyses (as δ^13^C). We show that oyster families selectively bred for fast growth and families selected for disease resistance can alter their mechanisms of calcite crystal biomineralization, promoting resilience to acidification. The responses of *S. glomerata* to acidification in their estuarine habitat provide key insights into mechanisms of mollusc shell growth under future climate change conditions. Importantly, we show that selective breeding in oysters is likely to be an important global mitigation strategy for sustainable shellfish aquaculture to withstand future climate‐driven change to habitat acidification.

## Graphical abstract

We characterized the crystallography and carbon uptake in the shells of the oyster *Saccostrea glomerata* being farmed in habitats subjected to acidification from land run‐off using high‐resolution electron backscatter diffraction and carbon isotope analyses (δ^13^C). Environmental carbon was linked with the changing carbon in the shell and differences in shell growth and crystallography across oyster families selectively bred for fast growth or disease resistance. We show that these oyster families can alter their mechanisms of biomineralization, promoting resilience to coastal acidification. Selective breeding in oysters is likely to be an important global mitigation strategy for sustainable aquaculture.

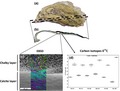

## 1 INTRODUCTION

Biomineral production by marine calcifiers is vulnerable to coastal acidification driven by land run‐off and rising sea level (Duarte et al., 2013; Fitzer et al., 2018) in addition to the more frequently investigated carbon dioxide (CO_2_)‐driven ocean acidification, with sensitivity varying between species. To understand this variability, and to evaluate potential solutions to compensate for the increased energy demands required to cope with these stressors (Parker et al., 2013; Thomsen, Casties, Pansch, Körtzinger, & Melzner, 2013), we need to identify the source of carbon used in biomineral formation and the mechanism of carbon uptake (Raven et al., 2005).

In marine calcifiers, the carbonate ions (CO_3_^2−^) used for calcium carbonate (CaCO_3_) shell construction are derived from two principal routes (Marin, Luquet, Marie, & Medakovic, 2008), environmental carbon and metabolic carbon. In bivalves, environmental carbon such as dissolved inorganic carbon (DIC) can be sourced in the form of CO_3_^2−^ or hydrogen carbonate (HCO_3_^−^) from ambient seawater. Acidification driven by CO_2_ uptake impairs shell growth as pH and CaCO_3_ saturation are lowered, resulting in reduced carbonate available for biomineralization (Doney, Fabry, Feely, & Kleypas, 2009). In contrast, respiratory CO_2_ enters the extrapallial (shell) fluid by diffusion from mantle tissue (Marin et al., 2008). Molluscs can also source CO_2_ from seawater and hydrolyse it to form HCO_3_^−^ in a process catalysed by carbonic anhydrase (Nicol, 1960; Roleda, Boyd, & Hurd, 2012; Wilbur, 1972), a highly conserved enzyme that functions in CO_2_ regulation. In mussels (*Mytilus edulis*) reared under CO_2_‐induced acidification, carbonic anhydrase activity is reduced (Fitzer, Phoenix, Cusack, & Kamenos, 2014; Fitzer, Vittert, et al., 2015; Fitzer, Zhu, et al., 2015). In addition to hydrolysed CO_2_ from seawater, HCO_3_^−^ from seawater and CO_3_^2−^ from tissues are also suggested to be sources of carbon for molluscan shell formation, which may differ depending on the environment (Nicol, 1960). For instance, HCO_3_^−^ is sourced directly from the surrounding water in the freshwater mollusc *Limnaea stagnalis* (Greenaway, 1971; Roleda et al., 2012). Where coastal acidification affects nearshore marine habitats due to freshwater run‐off (Duarte et al., 2013; Fitzer et al., 2018; Jiang et al., 2017), the changes to pH and DIC can alter carbonate availability which may limit biomineralization depending on the DIC used. Projected changes in carbonate saturation, as a result of environmental acidification from either mechanism (ocean or coastal) and in combination, are predicted to limit the ability of bivalves to produce their shells (Fitzer et al., 2014, 2018) endangering commercially important shellfish ventures in nearshore marine habitats.

In response to CO_2_‐induced acidification, bivalves may partition energy to maintain extracellular pH for the production of CO_3_^2−^ for calcification leaving less energy for shell growth (Gazeau et al., 2010; Parker et al., 2013). Increased metabolic rate under ocean acidification conditions is reported in *M. edulis*, the oyster *Saccostrea glomerata* and the clam *Laternula elliptica*, at pH 7.1–7.7, 7.9 and 7.7 respectively (Cummings et al., 2011; Gazeau et al., 2013; Parker et al., 2012, 2013; Thomsen & Melzner, 2010). This energy trade‐off of maintaining extracellular pH by increasing metabolism under decreased pH may be compensated for by increased food availability as seen for *M. edulis* and *S. glomerata* in CO_2_‐enriched environments (Parker et al., 2013; Thomsen et al., 2013).

Coastal acidification affects many nearshore marine habitats where freshwater run‐off results in reduced pH due to leachate from acid sulphate soils and humic acids and tannic acids from groundwater (Duarte et al., 2013; Fitzer et al., 2018; Jiang et al., 2017) and this is being exacerbated by climate change driven sea‐level rise and increasing catchment flooding and run‐off (Keene, Johnston, Bush, Burton, & Sullivan, 2010; Wong et al., 2010). This form of environmental acidification differs in chemistry mechanisms compared to ocean acidification (see Fitzer et al., 2018). Briefly, in CO_2_‐induced ocean acidification carbonic acid production alters the environmental DIC through reduced CO_3_^2−^ availability (Duarte et al., 2013; Fitzer et al., 2018; Jiang et al., 2017). In contrast, acidification in many coastal areas is driven by oxidation reactions in sulphate soils that produce sulphuric acid, a mineral acid that lowers total alkalinity (Duarte et al., 2013; Fitzer et al., 2018; Jiang et al., 2017), as also the case for humic and tannic acids. This form of environmental acidification also reduces dissolved oxygen caused by organic matter mineralization. Acidification in the coastal embayments used to culture shellfish is far more complex than the commonly investigated CO_2_‐driven ocean acidification. We investigate this for oysters resident in habitats prone to coastal acidification.

The orientation of the CaCO_3_ crystals is a key and ancient feature of bivalve shells with the standard or normal condition being highly ordered in organization (Marin, Roy, & Marie, 2012; Marin et al., 2008). In aragonite or nacreous shells, the tablets have their *a*, *b* and *c* axis co‐orientated with the *c* axis perpendicular to the aragonite surface and *b* axis parallel to the growth direction forming aragonite tablet layers (Marin et al., 2012). In calcite shells the prisms are orientated perpendicular to the outer shell (Marin et al., 2012). Deviations from the highly ordered crystal state are considered to indicate impaired or irregular biomineralization and importantly are associated with mechanically weaker shells, thus compromising their protective function. Such deviations have been observed in mussels and oysters reared in the laboratory under ocean acidification scenarios (Beniash, Ivanina, Lieb, Kurochkin, & Sokolova, 2010; Fitzer et al., 2014; Fitzer, Vittert, et al., 2015; Fitzer, Zhu, et al., 2015; Meng et al., 2018). Many ocean acidification studies show reduced shell growth, reduced shell thickness and mechanically weaker shells for molluscs growing in these conditions (Beniash et al., 2010; Dickinson et al., 2012; Fitzer et al., 2018; Fitzer, Vittert, et al., 2015; Fitzer, Zhu, et al., 2015; Gazeau et al., 2007; Meng et al., 2018; Ries, 2011; Ries, Cohen, & McCorkle, 2009). Interestingly, a similar disordered shell crystallography was observed in juvenile *Magallana angulata* grown under experimental acidification (CO_2_‐dosing; Meng et al., 2018) and in adult *S. glomerata* cultured in coastal acidified environments (natural pH gradient; Fitzer et al., 2018). In both ocean (e.g. CO_2_ uptake, upwelling) and coastal (low pH estuarine sediments) acidification, biomineralization is limited due to reduced availability of carbonate for shell production and energetic constraints (Barton et al., 2015; Byrne & Fitzer, 2019; Doney et al., 2009; Ekstrom et al., 2015; Gazeau et al., 2007, 2010; Parker et al., 2013).

Oyster aquaculture, the major component of a $19 billion mollusc aquaculture industry (FAO, 2016), is vulnerable to climate change‐driven acidification onshell growth due to global (e.g. atmospheric CO_2_ uptake) and local (e.g. land run‐off) stressors. The Sydney rock oyster, *S. glomerata*, forms the basis of a large aquaculture industry in coastal and estuarine locations in south‐eastern Australia (O'Connor & Dove, 2009). We investigated the impact of coastal acidification on biomineralization in this species in important oyster‐growing estuaries (Port Stephens, Wallace Lake) that are also known to be impacted by sulphate soil runoff (Dove & Sammut, 2013; O'Connor & Dove, 2009). In these areas, the production of *S. glomerata* has declined over recent decades attributed to water quality issues, including acidification from land run‐off and freshwater input (Dove & Sammut, 2013; Fitzer et al., 2018) as well as disease. Low total alkalinity has been noted in Wallis Lake (Fitzer et al., 2018). In both estuaries, oysters growing at the acidified sites regularly experience low pH (~pH 7.4–7.5; Fitzer et al., 2018). There has also been a decline in production of larger, higher value, ‘plate’ grade oysters and an increase in the smaller ‘bistro’ and ‘bottle’ grade oysters (O'Connor & Dove, 2009).

We used a novel approach to study the impact of natural acidification on biomineral pathways in *S. glomerata* being farmed in complex coastal habitats across sites differing in pH due to freshwater run‐off of humic acids and sulphate soils and incorporating natural fluctuations in salinity, temperature and biological production. This contrasts with the laboratory experimental acidification approach and so provides an assessment of the impacts of real‐world contemporary coastal acidification, an urgent problem around the globe being exacerbated by climate change. We characterized the crystallography and carbon uptake in the shells of *S. glomerata* being farmed in habitats subjected to acidification from land run‐off using high‐resolution electron backscatter diffraction (EBSD) and carbon isotope analyses (as δ^13^C). We use δ^13^C to link environmental carbon with the changing carbon in the shell and, therefore, differences in shell growth and crystallography across oyster families with respect to environment.

We availed of *S. glomerata* from families selectively bred for fast growth or disease resistance in the first study to assess whether this selection is also associated with changes in the mechanisms of calcite crystal biomineralization compared with that in wild‐type oysters. The larvae of these families exhibit better shell growth and development compared to wild‐type oysters under experimental acidification (Parker, Ross, & O'Connor, 2011; Parker et al., 2012). It is not known if this resilience of shell growth to acidification is also evident in the adult life stage and if this is due to a change in the biomineralization process. In consideration of the disrupted crystalline organization of the shells of bivalves reared in experimental acidification (Fitzer, Vittert, et al., 2015; Fitzer, Zhu, et al., 2015; Meng et al., 2018) and the disordered crystallography seen for *S. glomerata* families being cultured in coastal acidification estuaries (Fitzer et al., 2018), we hypothesized that there would be a change in δ^13^C incorporation into the shell in coastal *S. glomerata*. Based on differences in calcification of the larvae of the same families of *S. glomerata* reared in ocean acidification conditions (Parker et al., 2011, 2012), we expected to see similar differences in the ability to cope with acidification in the adult life stage. The complex coastal acidification in the estuarine habitat where these oysters are farmed, and their biomineralization response, provides insights into potential future changes to the mechanisms of mollusc shell growth under changing climates.

## 2 MATERIALS AND METHODS

Specimens of *S. glomerata* from families generated by the Sydney rock oyster breeding programme were obtained from commercial leases in Wallis Lake (Upper Wallamba latitude −32.174205, longitude 152.469004) and Port Stephens (Tilligerry Creek latitude −32.76852, longitude 151.965973). These major oyster‐growing estuaries were chosen as they are known to be influenced by sulphate soil acidification as well as having control and acidified sites (Dove & Sammut, 2013; Fitzer et al., 2018; O'Connor & Dove, 2009). Oysters (2015 year class, 1.5 years old, mean = 54.2 mm shell length, *SE* = 6.28 mm, *n* = 12) from families selectively bred for QX disease resistance (F15), fast growth on the basis of whole oyster weight (F30) and wild‐type (F31) bred from wild unselected parents were used. All families were compared at the control site and acidified site in Port Stephens. Additionally, the wild‐type family (F31) was also compared at a control and an acidified site in Wallis Lake. Of the 12 oysters sampled, six were randomly selected for the analyses, three for scanning electron microscopy (SEM) analyses and three for isotope analyses.

Tilligerry Creek, Port Stephens is a low‐lying floodplain containing a drainage network which discharges into Port Stephens estuary through a catchment area (130 km^2^) containing disturbed acid sulphate soils (Dove & Sammut, 2007). In Port Stephens the control site was Cromarty Bay. The sites in Wallis Lake included a ‘control’ site at Cockatoo Island, with estuarine salinities and the Upper Wallamba ‘acidified site’, a site which receives run‐off from identified areas of acid sulphate soil in the Wallamba River and has poor oyster growth.

### 2.1 Carbonate chemistry

Water samples were collected in triplicate at the time of oyster collection for each of the four sites (Table 1). Temperature, salinity and pH were measured on site using a pH probe calibrated on the total pH scale. Total alkalinity was analysed later using standard semiautomated titration, combined with spectrometric analysis using bromocresol indicator (Fitzer et al., 2018; Fitzer, Vittert, et al., 2015; Fitzer, Zhu, et al., 2015). The data from these snapshot samples (Table 1) are commensurate with data from long‐term monitoring (see details in Fitzer et al., 2018). The carbon chemistry at the acidified sites differs from the control, indicated by a reduced total alkalinity, reduced carbonate, aragonite and calcite saturation and an increase in *p*CO_2_ (Table 1).

**Table 1:** Estuarine water parameters for each oyster sampling site. The salinity, temperature, pH and total alkalinity were measured in triplicate from the oyster sampling sites at the time of oyster collection and used to calculate the carbonate chemistry parameters using CO2SYS in the total pH scale. Mean values for chlorophyll *a*, fDOM (a measure of tannins) and dissolved oxygen are based on long‐term monitoring data (see details in Fitzer et al., 2018)

Site	Salinity (ppt)	Temperature (°C)	pH	δ^13^C (‰)	Total alkalinity (µmol/kg)	CO_3_^2−^ (µmol/kg)	ΩCa	ΩAr	*p*CO_2_ (µatm)	Probe chlorophyll *a* (µg/L)	Probe fDOM (RFU)	Probe fDOM (QSU)	Probe DO %
Wallis lake, Cockatoo Island ‘Control’	33.7	20.4	8.21	1.34 ± 0.09	2,199 ± 3	233.6	5.64	3.66	242.2	3.0 ± 2.4	1.7 ± 0.9	2.4 ± 2.2	95.7 ± 2.4
Wallis lake, Upper Wallamba ‘Acidified site 1’	16.1	20.0	7.45	−6.28 ± 0.27	1,472 ± 13	21.2	0.58	0.35	1,407.1	5.3 ± 2.5	9.7 ± 13.5	30.3 ± 41.2	90.3 ± 14.5
Port Stephens Cromarty Bay (152.06221/−32.72295)	32.0	18.0	8.10	−1.10 ± 0.09	2,024 ± 9	162.1	3.95	2.54	302.6	1.7 ± 0.9	2.4 ± 2.2	7.9 ± 6.8	95.7 ± 2.4
Port Stephens, Tilligerry Creek ‘Acidified site 2’	26.6	17.8	7.81	−4.88 ± 0.12	1,897 ± 5	74.3	1.87	1.18	656.7	7.2 ± 2.7	17.2 ± 5.4	52.8 ± 16.5	85.8 ± 7.9

Comprehensive water chemistry and measurements of estuarine acidification from long‐term monitoring of the sites are detailed in Fitzer et al. (2018).

Dissolved inorganic carbon was measured in triplicate for seawater sampled and reported as δ^13^C VPDB (per mil; Scottish Universities Environment Research Centre, SUERC).

### 2.2 Oyster shell preparation for scanning electron microscope–electron backscatter diffraction (SEM–EBSD)

**Figure 1:**
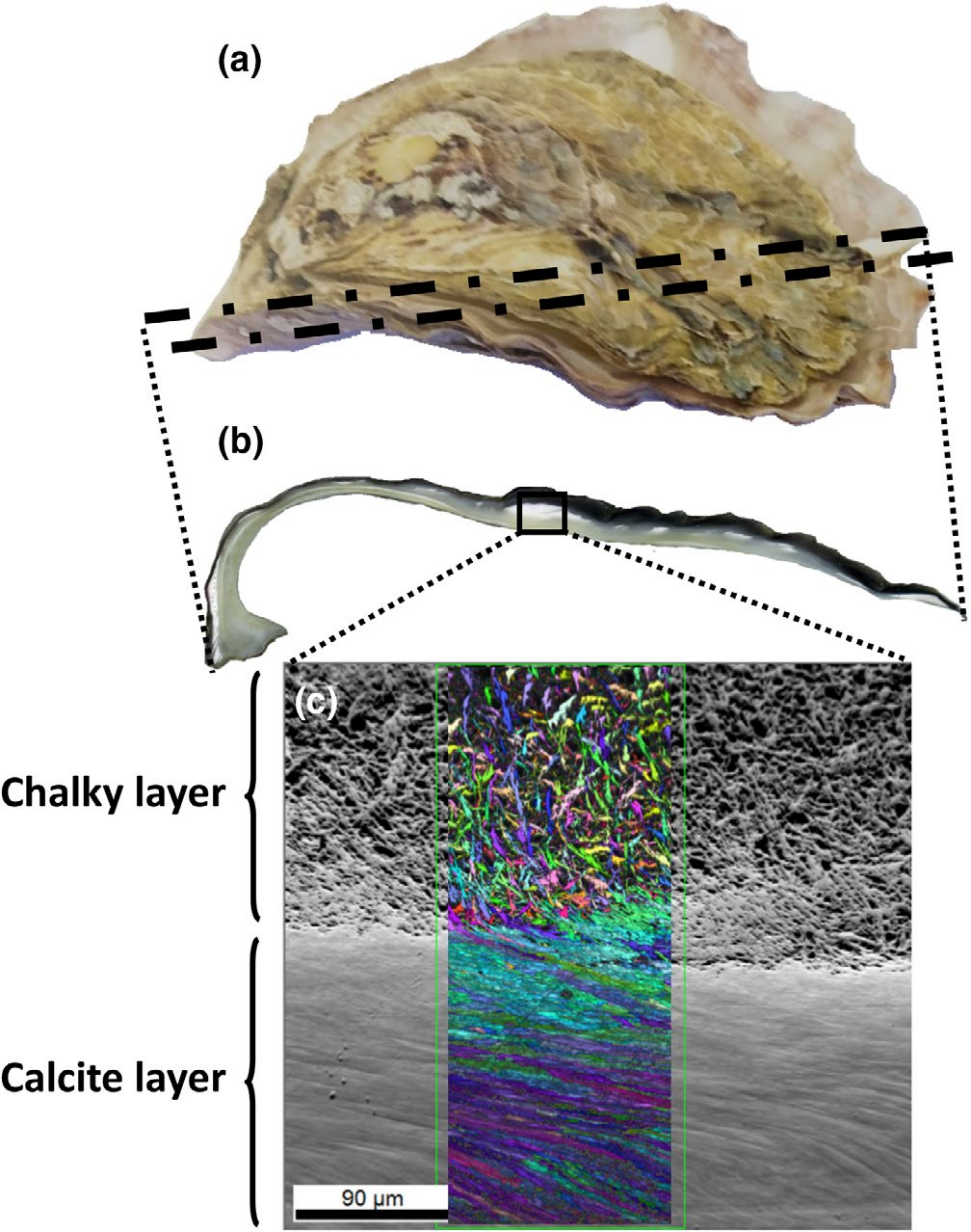
Schematic representation of the shell preparation for scanning electron microscope–electron backscatter diffraction (SEM–EBSD) showing the cut section (b) of the oyster shell (a) and area of imaging using SEM–EBSD (c). The chalky (white section of the shell) and calcite (the pearlescent section of the shell) layers can be distinguished and structural differences are presented in the SEM back‐scatter image (c)

**Figure 2:**
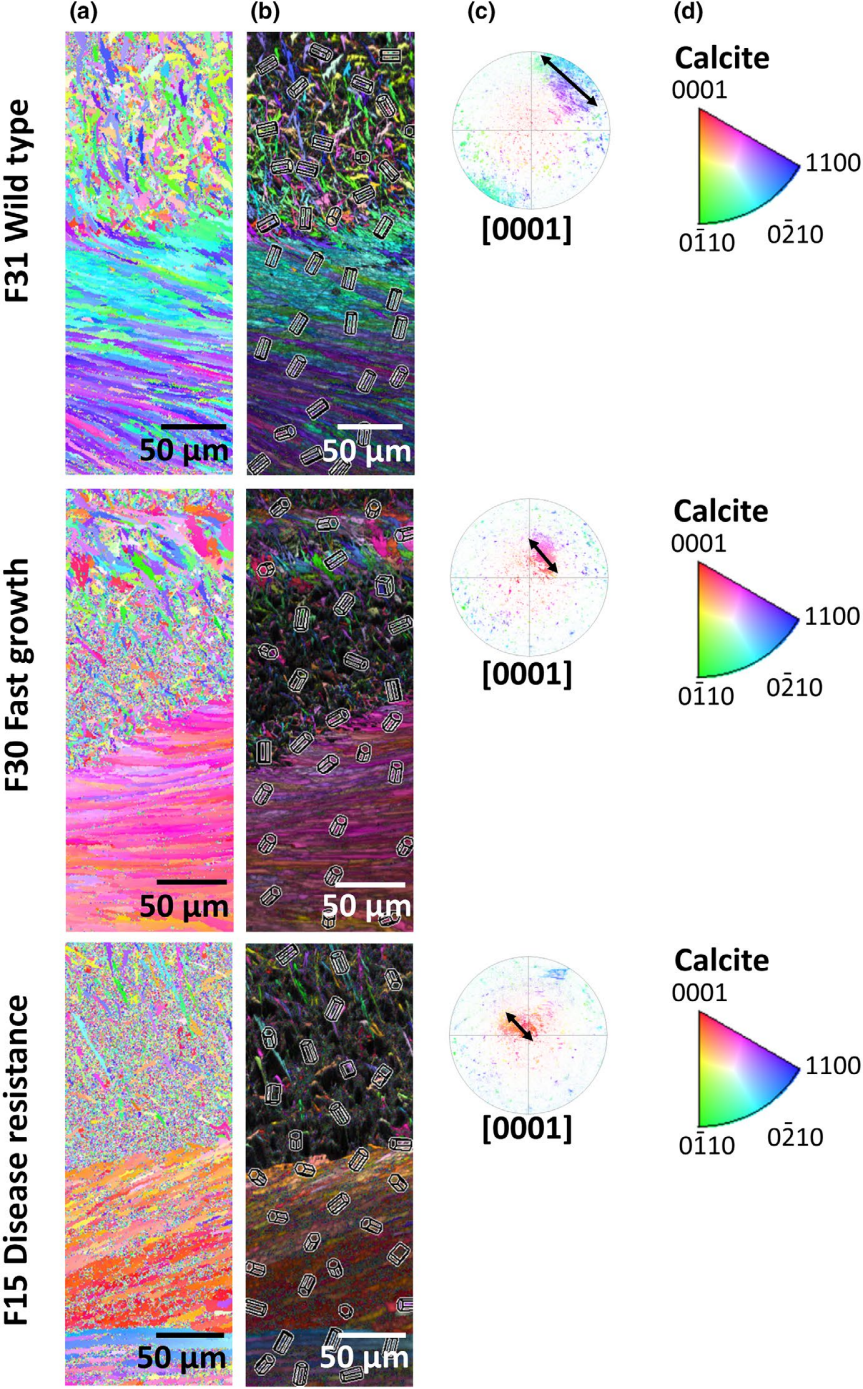
Summary of the SEM–EBSD data for wild‐type (F31), fast growth (F30) and disease resistance (F15) oyster families. (a) Crystallographic orientation maps for each family according to the colour keys (d) for calcite [0001]. (b) Crystallographic orientation map overlaid on the image quality of the SEM crystal structure with crystal lattices indicating the direction of the orientation of the crystal highlighted. (c) Inverse pole figures with 90° gridlines showing the crystallographic orientation data as per images (a). For each family, the clustering of the data, highlighted by an arrow, indicates the spread of the crystallographic orientation data for calcite as per the colour keys (d)

Oysters were dissected, shells rinsed with freshwater and air‐dried. The left ‘cupped’ valve was embedded in epoxy resin, sliced longitudinally with a diamond trim slow saw and polished for SEM (Fitzer et al., 2018; Figure 1). Briefly, the exposed cut shell section was polished using grit papers (P320, P800, P1200, P2500 and P4000), using polishing cloths with alpha alumina 1 µm and alpha alumina 0.3 µm, and finally using colloidal silica for 1 hr using a Vibromat. Shells were finished with distilled water and 10% methanol. Polished shell sections were analysed using SEM combined with EBSD for three individual oysters. The same midsection of the shell from the outer calcitic layer to the inner chalky layers was selected for each individual. Specimens were examined under low vacuum mode (~50 Pa) with an accelerating voltage of 20 kV using a FEI Quanta 200 F Environmental SEM with the stage tilted to 70° to examine backscatter Kikuchi patterns (Perez‐Huerta & Cusack, 2009) at the Imaging Spectroscopy and Analysis Centre (ISAACs), University of Glasgow, UK. Crystallographic orientation maps were produced through OIM Analysis™ V 7.0 software. EBSD collects quantitative crystallographic orientation data for each crystal point in an image. In the figures, each crystallographic orientation angle is highlighted by a colour with an accompanying colour key and also displayed on a pole figure (Figure 2). Pole figures show the 360° spread of crystallographic orientation data. The wider the spread for the data, the less ordered the crystallography.

### 2.3 Oyster shell carbon isotopes

Organic and inorganic δ^13^C analyses were conducted at the Isotope Geoscience Unit of the Scottish Universities Environmental Research Centre (SUERC). Oyster mantle tissue and extrapallial fluid were prepared for analysis by freeze‐drying the tissue and homogenizing using a pestle and mortar. Each sample was weighed using a microbalance and provided 0.7–1 mg of mantle tissue or extrapallial fluid powder for δ^13^C analysis via continuous flow isotope ratio mass spectroscopy (Elementar vario‐Pyrocube, Elemental Analyser interfaced with a Thermo Fisher Scientific, Delta Plus XP, Mass Spectrometer). Inorganic shell δ^13^C samples were prepared by micromilling 2–4 mg of calcite from the internal shell layer. The inorganic powder was plasma ashed (Emitech K1050X) overnight to remove any trace of organic tissue. The inorganic powder was then analysed for three individual oysters using an AP 2003 continuous flow automated carbonate system giving results as VPDB δ^13^C (Vienna Peedee Belemnite) Marine Carbonate Standard obtained from a Cretaceous marine fossil, *Belemnitella americana*.

### 2.4 Statistical analyses

A comparison of the seawater, shell, extrapallial fluid and mantle tissue carbon isotope data across families, environments and estuaries was made using a general linear model (GLM, Nelder & Wedderburn, 1972) with family (Port Stephens F15, F30, F31, Wallace Lake F31) and environment (control vs. acid) as factors. The full GLM was used to test whether oyster family population has an effect on the δ^13^C within the oyster and were run with family, environment (control vs. acid) and estuary (Port Stephens vs. Wallis Lake) as fixed factors (Table S5). In addition, a GLM was used to test whether the oyster family has an effect on the δ^13^C within the calcite, mantle tissue and extrapallial fluid at the Port Stephens estuary alone where all families were reared, run with family and environment (control vs. acid) as fixed factors (Table S6). GLM examines the iteratively weighted linear regression to obtain maximum likelihood estimates of the parameters with observations distributed according to some exponential family and systematic effects that can be made linear by a suitable transformation. A generalization of the analysis of variance is given for these models using log‐likelihood (Nelder & Wedderburn, 1972). Seawater δ^13^C from control and acidified sites in Port Stephens and Wallis Lake was compared using a two‐way ANOVA with environment (control vs. acid) and estuary (Port Stephens vs. Wallis Lake) as fixed factors. Assumptions of normality and homogeneity of variances were met, tested using probability distribution and a normal probability plot of residuals. All analyses were done using Minitab V18 (Minitab, Inc. http://www.minitab.com).

## 3 RESULTS

### 3.1 Estuarine acidification

**Figure 3:**
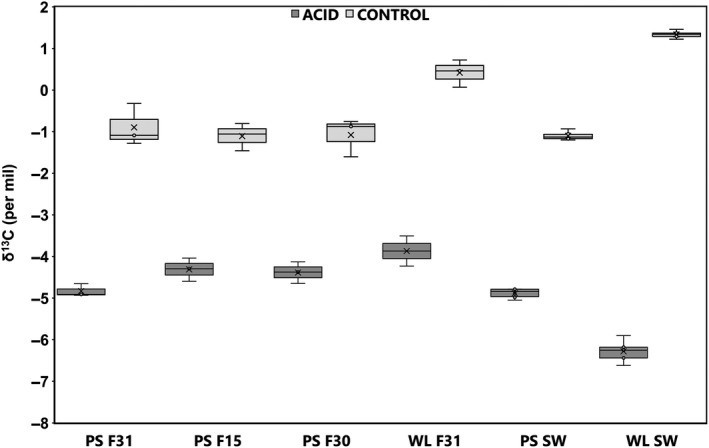
Shell calcite inorganic δ^13^C for wild‐type (F31), fast growth (F30) and disease resistance (F15) oyster families from control and acidified sites in Port Stephens (PS) and Wallis Lake (WL). Seawater (SW) δ^13^C is presented in comparison to the shell carbon for the control and acidified (acid) sites at PS and WL. Data are mean ± *SD* (*N* = 3)

The acidification of the two estuaries (Wallis Lake and Port Stephens) where the oysters are being farmed is complex with a marked difference in pH reflecting the well‐documented sulphate soil acidification in these estuaries (Dove & Sammut, 2013; Fitzer et al., 2018; O'Connor & Dove, 2009; Table 1). The increasing *p*CO_2_ is driven by freshwater‐induced acidification. As these naturally variable field sites are also influenced by salinity, temperature, dissolved oxygen (DO) and other biological factors (Table 1), it is not possible to statistically tease out the influence of these factors. Salinity decreases at both estuaries correlated with the reduced pH and increased *p*CO_2_. In parallel, chlorophyll *a* and fDOM increase suggesting an increase in biological production. The reduced pH and increased *p*CO_2_ is also accompanied by significantly lighter δ^13^C (GLM: site, *F*_1,21_ = 1.37, *p* = .257; environment, *F*_1,21_ = 155.41, *p* < .001; Table 1, Figure 3). The two acidified sites differed in pH at the time of sampling but both experience similar low pH levels (pH 7.4–7.5; see Fitzer et al., 2018). The salinity and temperature of the Wallis Lake versus Port Stephens estuaries was 33.7, 32.0 ppt and 20.4, 18.0°C, respectively. The δ^13^C in the seawater is significantly lighter in the acidified environment (control vs. acid), but does not differ between the two estuaries as shown by comparison of the response of families being farmed in the two regions (Wallis Lake vs. Port Stephens; GLM: estuary, *F*_1,21_ = 1.37, *p* = .257; environment, *F*_1,21_ = 155.41, *p* < .001). At the time of sampling, the control sites at Wallis Lake and Port Stephens had a pH of 8.2 and 8.1 (Table 1). The overall similarity in these parameters at the control sites is also evident from long‐term monitoring data (see Fitzer et al., 2018 Supporting Information).

### 3.2 Oyster shell crystallography

The shells of the *S. glomerata* are comprised of calcite prisms and chalky layers that produced good quality electron backscatter patterns (EBSP) during electron backscatter diffraction data acquisition (Figure 1). Shell crystallography shows clear differences between the wild type (F31) oyster families and those that have undergone selection for fast growth (F30) and disease resistance (F15; Figure 2; Figures S1–S3). The data shown on the crystallographic orientation maps for each family (Figure 2a) indicate differences in crystallographic orientation in the shells of the different families, where changes in the colour of crystallographic orientation are highlighted (see colour key Figure 2d). The crystal lattices (Figure 2b) indicate that the orientation of the crystals within the shell highlights an increased disorder in the crystallographic orientation in the wild‐type (F31) compared to the calcite layer in the shells of the fast growth (F30) and disease resistance (F15) families. The data from the maps are presented on crystallographic pole figures (Figure 2c). These pole figures show differences in the spread of crystallographic orientation data. The shells of wild‐type oysters appear more disordered than shells from the other families as indicated by the increase in the spread of the data across the pole figure (Figure 2c; Figure S3). In contrast, the crystallographic orientation data of the selected oyster families are clustered in the centre of the pole (Figure 2c). The data for the shells of the disease resistance family appear to be the most ordered with respect to control over crystallographic orientation of shell growth as seen in the clustering of the data at the centre of the pole figure and less spread of the data across the pole.

### 3.3 Oyster shell carbon isotopes

Oysters selectively bred for faster growth and disease resistance appear better able to cope with the lower carbon availability for shell growth in acidified conditions compared to the wild‐type oysters. Shell carbon incorporation reflects the seawater carbon isotopes, potentially an issue for growth under conditions where freshwater input of DIC is also associated with acidification from sulphate soil run‐off. This links shell growth to the changes in carbon chemistry (Figure 3; Table S1). The shell δ^13^C was significantly lighter, with a negative δ^13^C, in those oysters grown under coastal acidification (GLM: environment, *F*_1,22_ = 447.87, *p* < .001) coinciding with a significantly lighter seawater δ^13^C (GLM: *F*_1,21_ = 155.41, *p* < .001; Table S4). Oyster family significantly affected the δ^13^C within the shell with Wallis Lake wild‐type oysters in comparison to the Port Stephens wild‐type oysters showing similar patterns for calcite, with significantly lighter shell δ^13^C (GLM: family, *F*_3,22_ = 9.49, *p* = .001) under coastal acidification.

**Figure 4:**
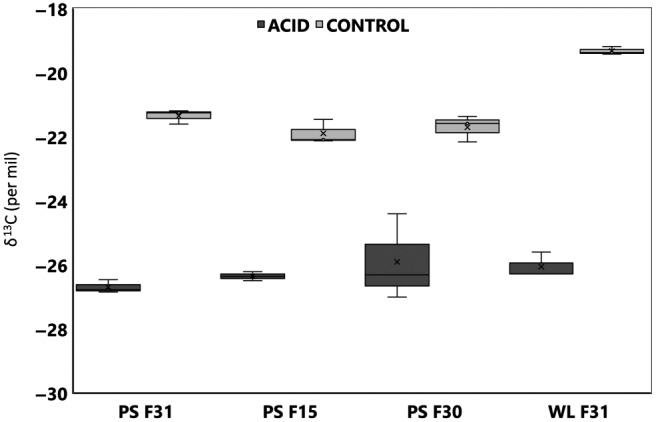
Mantle tissue δ^13^C for wild‐type (F31), fast growth (F30) and disease resistance (F15) oyster families from control and acidified sites in Port Stephens (PS) and Wallis Lake (WL). Seawater δ^13^C is presented in comparison to the shell carbon for the control and acidified (acid) sites at PS and WL. All isotope analyses were done in triplicate and error bars represent the mean ± *SD* (*N* = 3)

**Figure 5:**
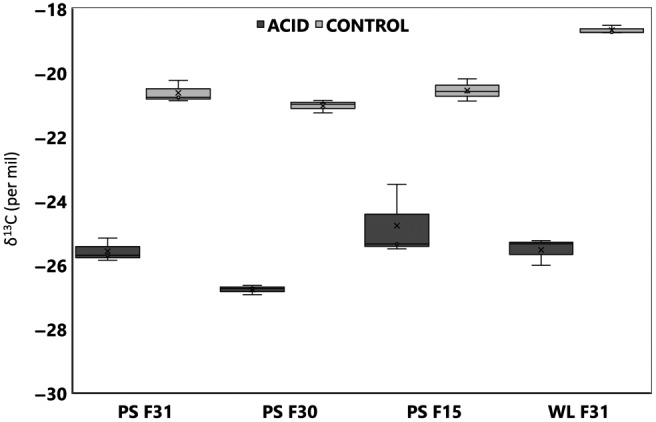
Extrapallial fluid δ^13^C for wild‐type (F31), fast growth (F30) and disease resistance (F15) oyster families from control and acidified sites in Port Stephens (PS) and Wallis Lake (WL). Seawater δ^13^C is presented in comparison to the shell carbon for the control and acidified (acid) sites at PS and WL. Data are mean ± *SD* (*N* = 3)

Tissues and extrapallial fluids are also affected by the lighter carbon in the seawater DIC (Figures 4 and 5; Tables S2 and S3). The mantle tissue (GLM: environment, *F*_1,23_ = 287.57, *p* < .001) and the extrapallial fluid (GLM: environment, *F*_1,23_ = 354.86, *p* < .001) δ^13^C was significantly lighter in those oysters grown under coastal acidification, coinciding with a significantly lighter seawater δ^13^C (GLM, environment, *F*_1,21_ = 155.41, *p* < .001; Table S4). The oysters have less control over the incorporation of heavier carbon in the form of carbonate to incorporate into the shell for growth.

The water from the Wallis Lake control site has a heavier δ^13^C compared to the acidified site in this estuary. In parallel, the shell inorganic δ^13^C follows a similar pattern providing more carbon to be available to the shell in control compared to the acidified sites when comparing with the Port Stephens wild‐type families (Figure 3). The resultant significant difference between δ^13^C in wild‐type shell growth under control compared to acidified conditions in Wallis Lake indicates a far greater freshwater influence over the lighter carbon incorporation at the low pH site (pH 7.45). When comparing the shell δ^13^C across families and environments specifically at the Port Stephens estuary alone, shell δ^13^C was significantly lighter, with a negative δ^13^C, in those oysters grown under coastal acidification (GLM: environment, *F*_1,17_ = 389.29, *p* < .001) coinciding with a significantly lighter seawater δ^13^C (ANOVA: *F*_1,11_ = 3,741.08, *p* < .001; Table S4). Oyster family did not significantly affect the δ13C within the shell (GLM: family, *F*_2,17_ = 0.31, *p* = .742; environment, *F*_1,17_ = 389.29, *p* < .001) or within the mantle tissue (GLM: family, *F*_2,17_ = 0.40, *p* = .678; environment, *F*_1,17_ = 247.77, *p* < .001) in comparison to the environment, however, in the extrapallial fluid the oyster family and the environment did have a significant effect on the δ^13^C (GLM: family, *F*_2,17_ = 6.49, *p* = .010; environment, *F*_1,17_ = 305.39, *p* < .001).

## 4 DISCUSSION

*Saccostrea glomerata* from families that were selectively bred for faster growth or disease resistance showed increased order in shell crystallography under coastal acidification compared to that for the shells of wild‐type oysters. A more ordered shell crystallography is indicative of an increased degree of structural order of the calcite which may lead to increased shell strength, as seen in mussels (Fitzer, Vittert, et al., 2015; Fitzer, Zhu, et al., 2015) and oysters (Ivanina et al., 2013). Similarly altered crystallographic orientation of mussel and oyster shells have been reported under acidification with increased disorder at pH 7.5, for *M. edulis* (Fitzer et al., 2014, 2018), *M. angulata* (Meng et al., 2018) and *S. glomerata* (Fitzer et al., 2018). This increase in shell crystallographic order indicates increased resilience to environmental acidification in *S. glomerata* selectively bred for faster growth or disease resistance compared with wild‐type oysters. These results parallel the better performance of the larvae of the selectively bred oysters reared under experimental acidification compared to the larvae of wild oysters, with respect to larval shell growth (Parker et al., 2011, 2012). The larvae generated from selectively bred *S. glomerata* had only a 24% reduction in shell growth in acidification conditions compared to a 64% reduction in wild populations (Parker et al., 2011). In addition, adults from the same selectively bred families also exhibited upregulation of calcification‐related genes under experimental acidification conditions, which may be indicative of their resilience to low pH (Goncalves et al., 2017). These results for the larval and adult life stages of *S. glomerata* suggest the presence of plastic or genetic compensatory mechanisms in the calcification response of selectively bred oysters in response to environmental acidification.

The two estuaries investigated here are complex coastal acidification environments. This presents a challenge in understanding the drivers that impact shell growth. Habitat acidification is driven by a variety of environmental factors associated with freshwater input, resulting in a parallel decline in pH and salinity, and an increase in *p*CO_2_ (Fitzer et al., 2018). The low pH levels point to the influence of acid sulphate floodplain drainage as well as run‐off of humic/tannic acids (Fitzer et al., 2018). Decreasing salinity can also be driven by enhanced input of humic acids from ground water as well as inputs of water from acid sulphate soils, also indicated by reduced DO indicative of oxidation reactions (Fitzer et al., 2018).

Environmental variations in salinity, temperature and biological productivity are known to impact oyster shell growth (Parker et al., 2017). The higher levels of chlorophyll *a* and fDOM at the low pH sites (Fitzer et al., 2018 Supporting Information) suggest an increase in biological production. This would be expected to increase shell growth due to enhanced food levels, but on the contrary, as seen here, the oysters at these acidified sites are characterized by slower shell growth rates (O'Connor & Dove, 2009). Thus, despite the potential for more food, increased *p*CO_2_ driven by coastal acidification is primarily responsible for altered shell growth at the crystal level, indicated by the δ^13^C (Table 1). As pH decreases and *p*CO_2_ increases, there is an accompanying lighter seawater δ^13^C of −6.28‰ (Wallis Lake) and −4.88‰ (Port Stephens). This same magnitude of lighter δ^13^C is observed in the shell of oysters grown in those acidified conditions. Therefore, for *S. glomerata*, coastal acidification appears to be the primarily driver of altered shell crystallography, rather than other potential factors such as salinity, temperature and biological productivity. That said, these factors may act as cofactors influencing the outcome with respect to shell growth. Temperature and salinity are important in controlling metabolic rate in oysters (Parker et al., 2017). In order to separate the influence of covarying factors such as pH and salinity controlled laboratory experiments are required, but it would be a challenge to emulate the complexity of the estuarine environment, though nonetheless key to teasing out mechanisms of action. In a previous experimentally controlled ocean acidification study, *S. glomerata* had a narrowed acute thermal and salinity tolerance as shown by lowered metabolic rate (Parker et al., 2017). It was suggested that the increased *p*CO_2_ led to constraints on aerobic performance, increasing energy demands when placed in lowered salinity (34.2–20 ppt) or increased temperature (+4°C) environments (Parker et al., 2017). Similar to this study on shell crystallography growth, acidification was suggested to impact the ability of the oyster to tolerate naturally occurring fluctuations in salinity and temperature.

The shell carbon isotope data suggest that there may be changes in the carbon source or biomineralization pathway of carbon uptake in *S. glomerata* grown under coastal acidification. Shell carbon isotope values were lighter, similar to that in the seawater that they were being farmed in. For shell carbonates, δ^13^C of less than −1‰ indicates that CO_3_^2−^ is the source of carbon, whereas between −1‰ and +1‰ indicates HCO_3_^−^ as a source of carbon (potentially metabolic; Grossman, 1984; Rohling & Cooke, 2003). The shells of *S. glomerata* grown under coastal acidification exhibited a lighter δ^13^C of less than −1‰, compared to those grown under control conditions irrespective of family selection or treatment. There was a change in the way that carbonate is incorporated into the shell in the wild‐type families, from HCO_3_^−^ as a source of carbon to CO_3_^2−^, compared to the fast growth and disease resistance families.

As typical of estuarine habitats, the seawater pH levels are naturally variable in Wallace Lake (pH 7.45–8.21) and Port Stephens (pH 7.84–8.08) due to freshwater input (Fitzer et al., 2018) and so it would be expected that the wild‐type *S. glomerata* would be adapted to cope well with such variability. However, the carbon in the shells of the wild‐type oysters from the control sites is between −1‰ and +1‰. This indicates HCO_3_^−^ is the source of carbon in near full seawater conditions. Under coastal acidification this changes to a δ^13^C of less than −1‰, indicating that CO_3_^2−^ is the source of carbon for shell formation in these oysters. This could be attributable to adaptation where the oyster selects HCO_3_^−^ as a source of carbon for shell growth through efficient use of carbonic anhydrase‐driven hydrolysis of seawater CO_2_ (Nicol, 1960; Roleda et al., 2012; Wilbur, 1972). In contrast, in the shells of the other families, the carbon was less than −1‰, δ^13^C, indicating that CO_3_^2−^ is the source of carbon at both the control and acidified sites. This difference in carbon source or biomineralization pathway of carbon incorporation between the wild type and QX disease resistance and fast growth families may explain why the oysters are able to control their crystallographic shell growth under acidification. While environmental acidification is the major influence driving shell δ^13^C dynamics, other factors such as food levels, metabolic rate and enzyme activity are also important (Marin et al., 2008; Nicol, 1960; Roleda et al., 2012; Wilbur, 1972). To tease out the effects of multiple parameters long‐term controlled experiments would be informative, albeit challenging.

The wild‐type oyster would be expected to be adapted for background variable freshwater‐driven pH fluctuations through efficient use of carbonic anhydrase activity and changing carbon source under extreme coastal acidification. Where coastal acidification can result in the reduced activity of carbonic anhydrase (e.g. *M. edulis*, Fitzer, Vittert, et al., 2015; Fitzer, Zhu, et al., 2015; *S. spallanzanii*, Turner, Ricevuto, Massa‐Gallucci, Gambi, & Calosi, 2015), this adaptation may be less favourable for shell growth under these conditions. In the coccolithophore, *Ochrosphaera neapolitana,* it was also suggested that in response to depleted seawater DIC (δ^13^C) under experimental ocean acidification, the carbon source is switched to an internal δ^13^C pool to maintain calcification (Liu, Eagle, Aciego, Gilmore, & Ries, 2018). It would appear that *S. glomerata*, similar to the coccolithophore, has a reduced HCO_3_^−^ uptake under the depleted seawater δ^13^C where the δ^13^C in the shell is less than −1‰ indicating a switch to CO_3_^2−^ under naturally occurring coastal acidification (Liu et al., 2018). Carbon uptake mechanisms, therefore, may be taxon specific, similarly to phenotypic plasticity in mussels, gastropods and planktonic copepods (Vargas et al., 2017). Determining carbon uptake mechanisms across a diversity of commercial shellfish species is important to understand their vulnerability to climate change and potential approaches to mitigation strategies.

## 5 CONCLUSIONS

This study is the first to identify differences in the mechanisms of shell growth attributable to selective breeding for an oyster species faced with coastal acidification in commercial leases. The resilience of the selected families of *S. glomerata*, highlights the potential for selective breeding to at least partially ameliorate the negative effects of climate change‐driven coastal acidification on oyster shell growth, as well as ocean acidification. We show the potential for selective breeding to provide a more resilient oyster for commercial aquaculture to withstand future climate change‐driven coastal acidification, in agreement with studies of the larval stage (Parker et al., 2011, 2012). Selective breeding may be an important mitigation strategy to climate proof the global shellfish aquaculture industries where coastal acidification is being exacerbated by climate change.

## CONFLICT OF INTEREST

There are no conflicts of interest for this submission.

## AUTHOR CONTRIBUTIONS

S.C.F. and M.B. designed and undertook field ecosystem experiments. S.C.F., M.B., S.T.G., B.H. and M.D. were all involved in the acquisition of field data. S.C.F. and R.M. analysed field samples and interpreted the microscopy data. S.C.F. interpreted the data and drafted the manuscript with M.S. S.C.F., M.B., R.M., B.H., M.D., and W.O. revising the work critically for important intellectual content and approving the final version for publication.

## Data Availability

All data for Figures S3–S5 are available in Tables S1–S4 of the Supporting Information.
