# Supplementary material for: Selectively bred oysters can alter their biomineralization pathways, promoting resilience to environmental acidification
Source: Glob Chang Biol. 2019 Sep 25;25(12):4105–15. doi: 10.1111/gcb.14818 (PMC6899863; doi:10.1111/gcb.14818)
Supplement: Supplementary file 1 [file GCB-25-4105-s001.docx]

**Supporting information for:**

**Selectively bred oysters can alter their biomineralization pathways, promoting resilience to environmental acidification**

S. C. Fitzer^1*^, R. A. R. McGill^2^, S. Torres Gabarda^3^, B. Hughes^4^, M. Dove^5^, W. O’Connor^5^, and M. Byrne^3,6**^

^1^ Institute of Aquaculture, Pathfoot Building, University of Stirling, Stirling, FK9 4LA, UK

^2^ Scottish Universities Environmental Research Centre, Rankine Avenue, Scottish Enterprise Technology Park, East Kilbride, G75 0QF, UK

^3^ School of Medical Sciences, University of Sydney, NSW 2006, Australia

^4^ Hunter Local Land Services, 98 Victoria St, PO Box 440, Taree 2430, Australia

^5^ New South Wales Department of Primary Industries, Fisheries NSW, Port Stephens Fisheries Institute, Taylors Beach Rd, Taylors Beach NSW 2316, Australia

^6^ School of Life and Environmental Sciences, University of Sydney, NSW 2006, Australia

Corresponding authors: [*susan.fitzer@stir.ac.uk](mailto:*susan.fitzer@stir.ac.uk), ** [maria.byrne@sydney.edu.au](mailto:maria.byrne@sydney.edu.au)


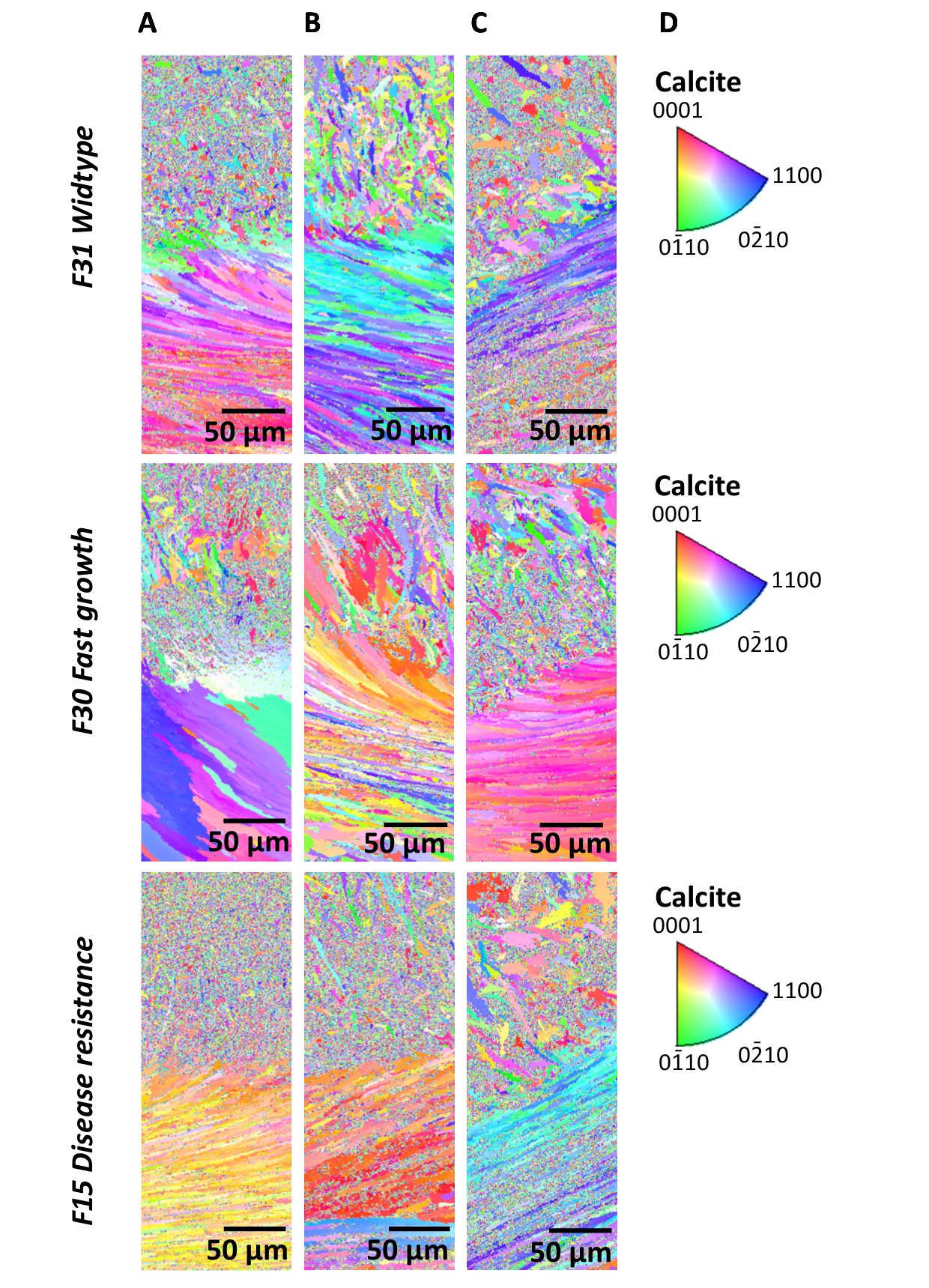


**Figure S1. Crystallographic orientation maps for three individual oysters (A, B, C) for each family line according to the colour keys (D.) for calcite [0001].**

**
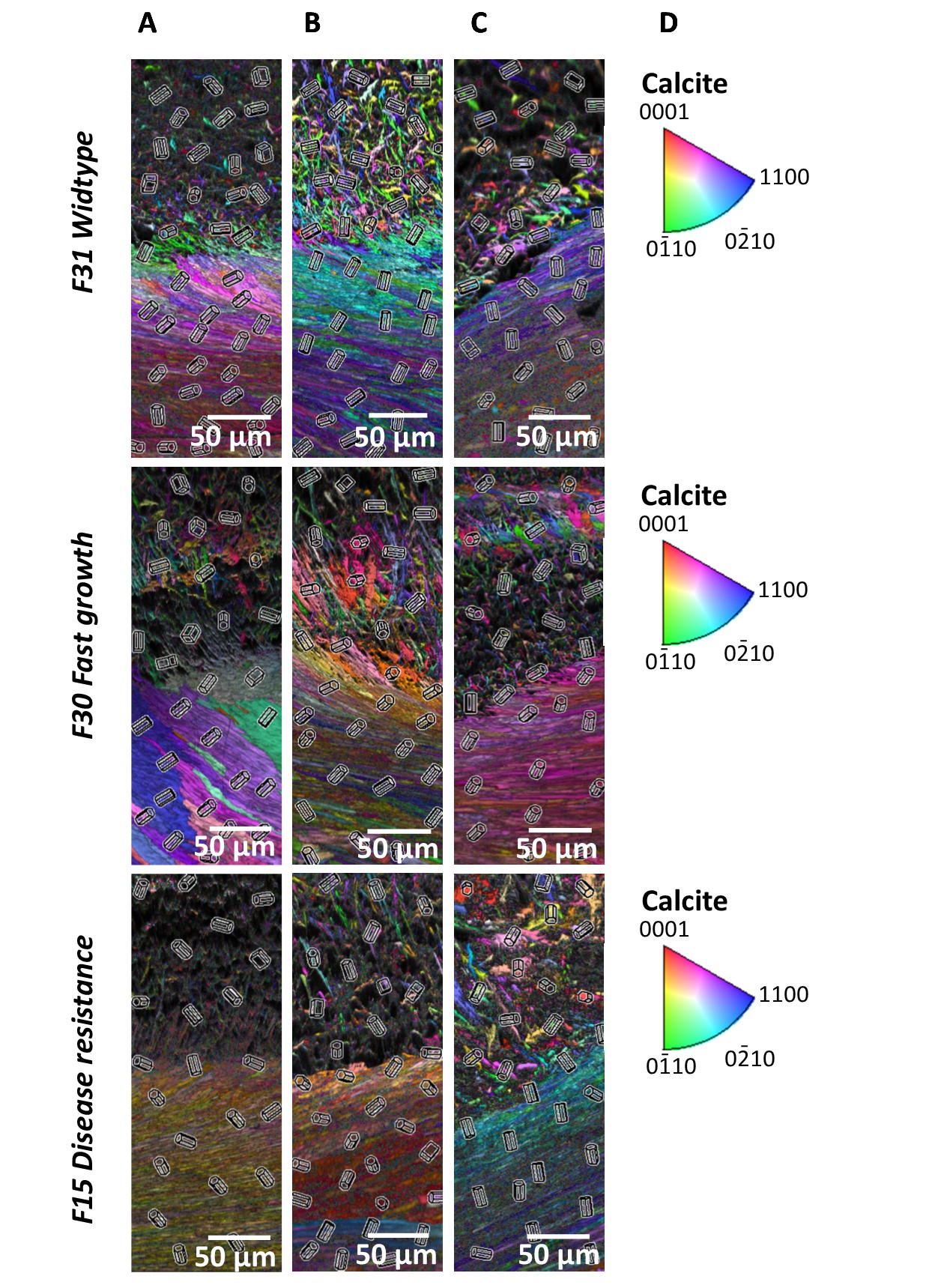
**

**Figure S2. Crystallographic orientation maps overlaid on the image quality of the SEM crystal structure with crystal lattices indicating the direction of the orientation of the crystal highlighted for three individual oysters (A, B, C) for each family line according to the colour keys (D.) for calcite [0001].**

**
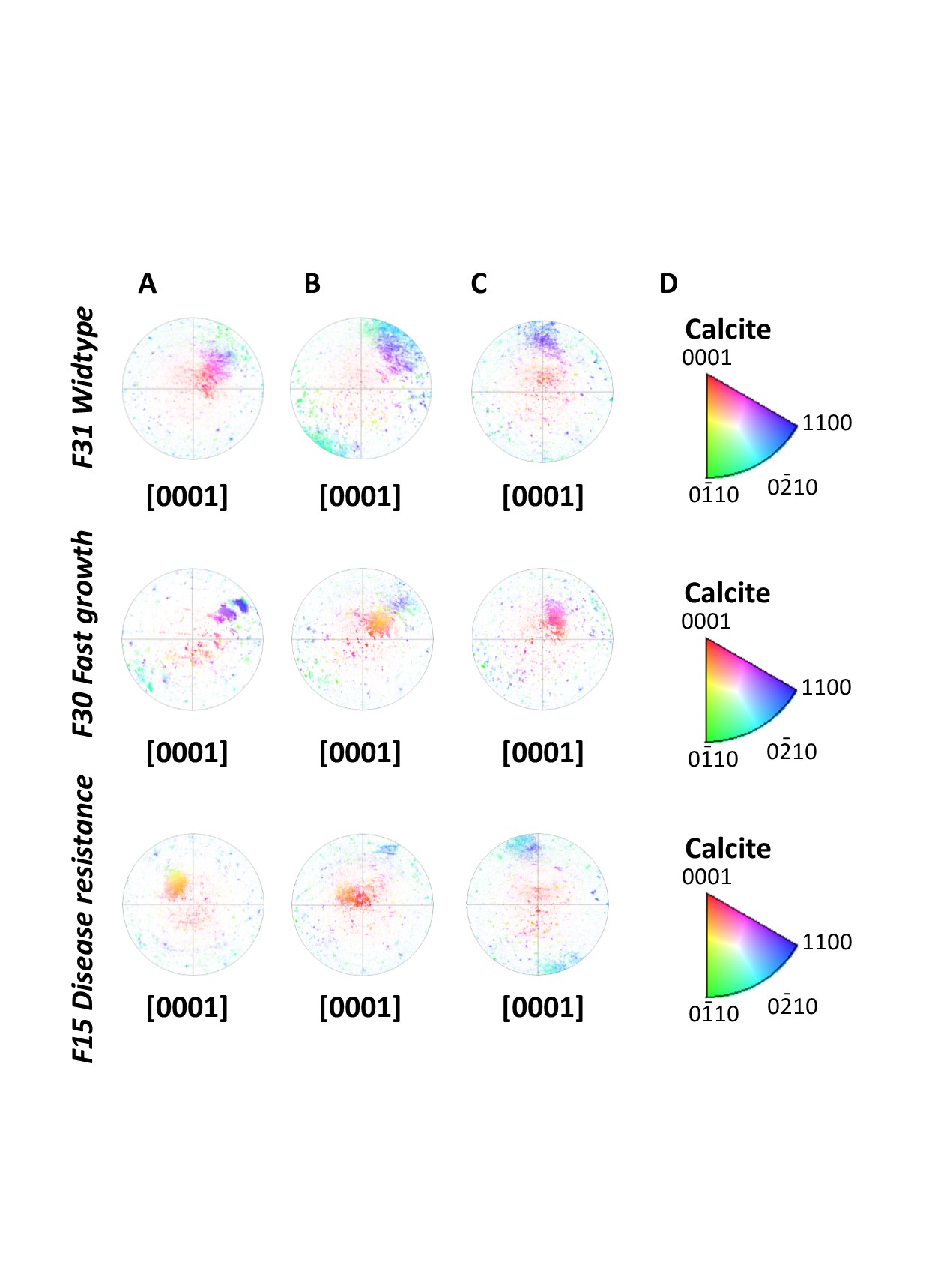
**

**Figure S3. Inverse pole figures showing the crystallographic orientation data as per images in supplementary figure 1 (A, B, C) for each family line, the clustering of the data indicates how spread the crystallographic orientation according to the colour keys (D.) for calcite [0001].**

**Table S1. Shell calcite inorganic δ^13^C for selected wildtype (F31), fast growth (F30) and disease resistance (F15) family lines from control and acidified sites in Port Stephens and Wallis Lake.**

| Field site | Growth line description | Family | Environment | δ^13^C VPDB |
| --- | --- | --- | --- | --- |
| Port Stephens | Wild | 31 | Control | -0.32 |
| Port Stephens | Wild | 31 | Control | -1.28 |
| Port Stephens | Wild | 31 | Control | -1.09 |
| Port Stephens | Wild | 31 | Acid | -4.91 |
| Port Stephens | Wild | 31 | Acid | -4.65 |
| Port Stephens | Wild | 31 | Acid | -4.93 |
| Port Stephens | Disease resistance | 15 | Control | -1.05 |
| Port Stephens | Disease resistance | 15 | Control | -0.80 |
| Port Stephens | Disease resistance | 15 | Control | -1.46 |
| Port Stephens | Disease resistance | 15 | Acid | -4.60 |
| Port Stephens | Disease resistance | 15 | Acid | -4.04 |
| Port Stephens | Disease resistance | 15 | Acid | -4.30 |
| Port Stephens | Fast | 30 | Control | -1.60 |
| Port Stephens | Fast | 30 | Control | -0.76 |
| Port Stephens | Fast | 30 | Control | -0.87 |
| Port Stephens | Fast | 30 | Acid | -4.13 |
| Port Stephens | Fast | 30 | Acid | -4.37 |
| Port Stephens | Fast | 30 | Acid | -4.65 |
| Wallis Lake | Wild | 31 | Acid | -4.23 |
| Wallis Lake | Wild | 31 | Acid | * |
| Wallis Lake | Wild | 31 | Acid | -3.51 |
| Wallis Lake | Wild | 31 | Control | 0.72 |
| Wallis Lake | Wild | 31 | Control | 0.07 |
| Wallis Lake | Wild | 31 | Control | 0.46 |

**Table S2. Mantle tissue organic δ^13^C for selected wildtype (F31), fast growth (F30) and disease resistance (F15) family lines from control and acidified sites in Port Stephens.**

| Field site | Tissue | Growth line | Family | Environment | δ^13^C VPDB |
| --- | --- | --- | --- | --- | --- |
| Port Stephens | Mantle | Wild | F31 | Acid | -26.47 |
| Port Stephens | Mantle | Wild | F31 | Acid | -26.85 |
| Port Stephens | Mantle | Wild | F31 | Acid | -26.78 |
| Port Stephens | Mantle | Wild | F31 | Control | -21.61 |
| Port Stephens | Mantle | Wild | F31 | Control | -21.19 |
| Port Stephens | Mantle | Wild | F31 | Control | -21.26 |
| Port Stephens | Mantle | Disease resistance | F15 | Acid | -26.22 |
| Port Stephens | Mantle | Disease resistance | F15 | Acid | -26.36 |
| Port Stephens | Mantle | Disease resistance | F15 | Acid | -26.50 |
| Port Stephens | Mantle | Disease resistance | F15 | Control | -22.09 |
| Port Stephens | Mantle | Disease resistance | F15 | Control | -22.13 |
| Port Stephens | Mantle | Disease resistance | F15 | Control | -21.46 |
| Port Stephens | Mantle | Fast | F30 | Acid | -26.32 |
| Port Stephens | Mantle | Fast | F30 | Acid | -24.41 |
| Port Stephens | Mantle | Fast | F30 | Acid | -27.01 |
| Port Stephens | Mantle | Fast | F30 | Control | -21.59 |
| Port Stephens | Mantle | Fast | F30 | Control | -22.16 |
| Port Stephens | Mantle | Fast | F30 | Control | -21.37 |

**Table S3. Mantle tissue organic δ^13^C for selected wild type (F31), fast growth (F30) and disease resistance (F15) family lines from control and acidified sites in Port Stephens.**

| Field site | Tissue | Growth line | Family | Environment | δ^13^C VPDB |
| --- | --- | --- | --- | --- | --- |
| Port Stephens | EP | Wild | F31 | Acid | -25.17 |
| Port Stephens | EP | Wild | F31 | Acid | -25.70 |
| Port Stephens | EP | Wild | F31 | Acid | -25.86 |
| Port Stephens | EP | Wild | F31 | Control | -20.77 |
| Port Stephens | EP | Wild | F31 | Control | -20.24 |
| Port Stephens | EP | Wild | F31 | Control | -20.88 |
| Port Stephens | EP | Fast | F30 | Acid | -26.94 |
| Port Stephens | EP | Fast | F30 | Acid | -26.74 |
| Port Stephens | EP | Fast | F30 | Acid | -26.65 |
| Port Stephens | EP | Fast | F30 | Control | -20.99 |
| Port Stephens | EP | Fast | F30 | Control | -21.26 |
| Port Stephens | EP | Fast | F30 | Control | -20.87 |
| Port Stephens | EP | Disease resistance | F15 | Acid | -23.49 |
| Port Stephens | EP | Disease resistance | F15 | Acid | -25.51 |
| Port Stephens | EP | Disease resistance | F15 | Acid | -25.35 |
| Port Stephens | EP | Disease resistance | F15 | Control | -20.59 |
| Port Stephens | EP | Disease resistance | F15 | Control | -20.20 |
| Port Stephens | EP | Disease resistance | F15 | Control | -20.89 |

**Table S4. Seawater δ^13^C from control and acidified sites in Port Stephens and Wallis Lake.**

| Field site | Seawater | δ^13^C VPDB |
| --- | --- | --- |
| Port Stephens | Acid | -4.89 |
| Port Stephens | Acid | -4.79 |
| Port Stephens | Acid | -5.05 |
| Port Stephens | Acid | -4.78 |
| Port Stephens | Acid | -4.98 |
| Port Stephens | Acid | -4.79 |
| Port Stephens | Control | -1.16 |
| Port Stephens | Control | -1.17 |
| Port Stephens | Control | -1.10 |
| Port Stephens | Control | -0.93 |
| Port Stephens | Control | -1.20 |
| Port Stephens | Control | -1.05 |
| Wallis Lake | Acid | -6.25 |
| Wallis Lake | Acid | -6.44 |
| Wallis Lake | Acid | -5.90 |
| Wallis Lake | Acid | -6.62 |
| Wallis Lake | Acid | -6.18 |
| Wallis Lake | Control | 1.29 |
| Wallis Lake | Control | 1.22 |
| Wallis Lake | Control | 1.34 |
| Wallis Lake | Control | 1.37 |
| Wallis Lake | Control | 1.46 |

**Table S5. General Linear Model (GLM) output with analysis of variance for shell (a), mantle tissue (b) and extrapallial fluid (c) δ^13^C versus estuary, environment and family, where QX disease resistance (F15), fast growth on the basis of whole oyster weight (F30) and wild type (F31) bred from wild unselected parents were used.**

**Factor Information for GLM design**

| Factor | Type | Levels | Values |
| --- | --- | --- | --- |
| Estuary | Fixed | 2 | Port Stephens, Wallis Lake |
| Environment | Fixed | 2 | Acid, Control |
| Family | Fixed | 3 | 15, 30, 31 |

1. **Analysis of variance of the GLM for shell δ^13^C**

| Source | DF | Adj SS | Adj MS | F-Value | P-Value |
| --- | --- | --- | --- | --- | --- |
| Estuary | 1 | 3.9207 | 3.9207 | 23.10 | 0.000 |
| Family | 2 | 0.0856 | 0.0428 | 0.25 | 0.780 |
| Environment | 1 | 76.0041 | 76.0041 | 447.87 | 0.000 |
| Error | 18 | 3.0546 | 0.1697 |  |  |
| Lack-of-Fit | 3 | 1.0853 | 0.3618 | 2.76 | 0.079 |
| Pure Error | 15 | 1.9693 | 0.1313 |  |  |
| Total | 22 | 87.6053 |  |  |  |

1. **Analysis of variance of the GLM mantle tissue δ^13^C**

| Source | DF | Adj SS | Adj MS | F-Value | P-Value |
| --- | --- | --- | --- | --- | --- |
| Estuary | 1 | 5.333 | 5.333 | 9.50 | 0.006 |
| Family | 2 | 0.317 | 0.158 | 0.28 | 0.757 |
| Environment | 1 | 161.414 | 161.414 | 287.57 | 0.000 |
| Error | 19 | 10.665 | 0.561 |  |  |
| Lack-of-Fit | 3 | 5.848 | 1.949 | 6.48 | 0.004 |
| Pure Error | 16 | 4.816 | 0.301 |  |  |
| Total | 23 | 179.937 |  |  |  |

1. **Analysis of variance of the GLM extrapallial fluid δ^13^C**

| Source | DF | Adj SS | Adj MS | F-Value | P-Value |
| --- | --- | --- | --- | --- | --- |
| Estuary | 1 | 2.994 | 2.994 | 5.98 | 0.024 |
| Family | 2 | 4.727 | 2.363 | 4.72 | 0.022 |
| Environment | 1 | 177.781 | 177.781 | 354.86 | 0.000 |
| Error | 19 | 9.519 | 0.501 |  |  |
| Lack-of-Fit | 3 | 5.758 | 1.919 | 8.16 | 0.002 |
| Pure Error | 16 | 3.761 | 0.235 |  |  |
| Total | 23 | 197.691 |  |  |  |

**Table S6. General Linear Model (GLM) output with analysis of variance for shell (a), mantle tissue (b) and extrapallial fluid (c) δ^13^C versus environment and family for Port Stephens estuary data alone, where QX disease resistance (F15), fast growth on the basis of whole oyster weight (F30) and wild type (F31) bred from wild unselected parents were used.**

**Factor Information for GLM design**

| **Factor** | **Type** | **Levels** | **Values** |
| --- | --- | --- | --- |
| Family | Fixed | 3 | F15, F30, F31 |
| Environment | Fixed | 2 | Acid, Control |

1. **Analysis of variance of the GLM for shell δ^13^C**

| **Source** | **DF** | **Adj SS** | **Adj MS** | **F-Value** | **P-Value** |
| --- | --- | --- | --- | --- | --- |
| Family | 2 | 0.0856 | 0.0428 | 0.31 | 0.742 |
| Environment | 1 | 54.5646 | 54.5646 | 389.29 | 0.000 |
| Error | 14 | 1.9623 | 0.1402 |  |  |
| Lack-of-Fit | 2 | 0.4718 | 0.2359 | 1.90 | 0.192 |
| Pure Error | 12 | 1.4905 | 0.1242 |  |  |
| Total | 17 | 56.6125 |  |  |  |

1. **Analysis of variance of the GLM mantle tissue δ^13^C**

| **Source** | **DF** | **Adj SS** | **Adj MS** | **F-Value** | **P-Value** |
| --- | --- | --- | --- | --- | --- |
| Family | 2 | 0.317 | 0.1585 | 0.40 | 0.678 |
| Environment | 1 | 98.217 | 98.2168 | 247.77 | 0.000 |
| Error | 14 | 5.550 | 0.3964 |  |  |
| Lack-of-Fit | 2 | 1.076 | 0.5378 | 1.44 | 0.275 |
| Pure Error | 12 | 4.474 | 0.3728 |  |  |
| Total | 17 | 104.083 |  |  |  |

1. **Analysis of variance of the GLM extrapallial fluid δ^13^C**

| **Source** | **DF** | **Adj SS** | **Adj MS** | **F-Value** | **P-Value** |
| --- | --- | --- | --- | --- | --- |
| Family | 2 | 4.727 | 2.363 | 6.49 | 0.010 |
| Environment | 1 | 111.140 | 111.140 | 305.39 | 0.000 |
| Error | 14 | 5.095 | 0.364 |  |  |
| Lack-of-Fit | 2 | 1.719 | 0.859 | 3.05 | 0.085 |
| Pure Error | 12 | 3.376 | 0.281 |  |  |
| Total | 17 | 120.961 |  |  |  |
